# Mucosal disease associated with BVDV-1b and BVDV-1e in cattle in Southern Brazil

**DOI:** 10.1007/s11250-026-05439-2

**Published:** 2026-10-23

**Authors:** Lucas Marian, Letícia F. Baumbach, Jéssica A. Withoeft, Anthony B. Ferreira, Monika Hilbe, Eduarda V. Rocha, Claudia S. Wisser, Ubirajara M. da Costa, Cláudio W. Canal, Renata A. Casagrande

**Affiliations:** 1 Laboratório de Patologia Animal, Centro de Ciências Agroveterinárias, Universidade do Estado de Santa Catarina (UDESC), Lages, Santa Catarina Brazil; 2 Laboratório de Virologia Veterinária, Universidade Federal do Rio Grande do Sul (UFRGS), Porto Alegre, Rio Grande do Sul Brazil; 3 Centro de Diagnóstico Microbiológico Animal (CEDIMA), Centro de Ciências Agroveterinárias, Universidade do Estado de Santa Catarina (UDESC), Lages, Santa Catarina Brazil; 4 Institute of Veterinary Pathology, Vetsuisse Faculty Zurich, University of Zuerich, Zuerich, Switzerland

**Keywords:** Ruminants, Virology, Pathology, Phylogeny

## Abstract

Bovine viral diarrhea virus (BVDV) is an important pathogen of the global cattle industry. Fetal infection during the period of immunotolerance may result in the birth of persistently infected (PI) animals. In such animals, the emergence of or superinfection with cytopathic variants may trigger mucosal disease (MD). The objective of this study was to characterize the anatomopathological and virological aspects of two cases of MD. Animal 1, a 24-month-old presented with diarrhea, weight loss, lameness, sialorrhea, and oral and cutaneous lesions. Animal 2, a four-month-old, exhibited weight loss, intermittent diarrhea, and dehydration. Both animals were submitted to necropsy, and tissue samples from all organs were collected for histopathological evaluation. For BVDV detection, serum samples were subjected to RT-PCR and amplified products were submitted to Sanger sequencing and phylogenetic analysis. Liver and spleen fragments were used for viral isolation in MDBK cell cultures. Immunohistochemistry using the panpestiviral monoclonal antibody 15C5 was performed to detect BVDV antigen. Anatomopathological examination of Animal 1 revealed ulcerative glossitis, erosive stomatitis, erosive and ulcerative esophagitis as well as erosive and ulcerative rumenitis, erosive abomasitis, and ulcerative and crusting dermatitis affecting the nasal planum and the distal limb region. In Animal 2, ulcerative rumenitis, erosive abomasitis, and lymphoid depletion in the ileal Peyer’s patches were observed. Both animals were positive for BVDV in immunohistochemistry and classified as BVDV-1e and BVDV-1b based on phylogenetic analysis. Viral isolates from both cases induced cytopathic effect between 48 and 96 h post-inoculation. This study demonstrates distinct lesion patterns in unrelated cases of MD associated with BVDV-1e and BVDV-1b in cattle, constitutes the first report of typical MD associated with these subgenotypes in Brazil.

## Introduction

In cattle production, diseases contribute to economic losses by reducing productivity (meat, milk, and reproductive performance) and limiting the ability of females to reach their genetic potential (Çilek et al. 2005; Çilek and Gotoh 2011). Among these, bovine viral diarrhea virus (BVDV) is one of the most significant pathogens, representing a major challenge to the global cattle industry and causing substantial productive and economic losses (Riley et al. 2019).

Bovine viral diarrhea virus (BVDV) belongs to the family *Flaviviridae* and the genus *Pestivirus*. According to recent taxonomic updates, the virus is classified as *Pestivirus bovis* (BVDV-1), *Pestivirus tauri* (BVDV-2), and *Pestivirus braziliense* (BVDV-3 or HoBi-like), all of which are capable of inducing subclinical, acute, chronic, and persistent infections, depending on strain virulence and host immune status (Postler et al. 2023). The virus is further categorized into cytopathic (CP) and non-cytopathic (NCP) biotypes based on its effect in cell culture (Zhang et al. 2026).

Viral epidemiology is predominantly driven by NCP strains, which play a critical role in maintaining infection within herds. This biotype is responsible for the generation of persistently infected (PI) animals, which arise following fetal infection between 40 and 120 days of gestation, prior to the development of fetal immunocompetence (Walz et al. 2020). PI animals are born immunotolerant and remain persistently viremic, acting as biological reservoirs and continuous sources of viral dissemination. These animals typically have a reduced life expectancy and are highly susceptible to secondary infections and progression to mucosal disease (MD) (Al-Kubati et al. 2021).

The term *mucosal disease* refers to a severe and often fatal manifestation of BVDV infection that results from superinfection with an antigenically homologous CP strain or, more commonly, from mutations arising in the resident NCP strain within a PI animal (Bianchi et al. 2017). The condition is characterized by erosive and ulcerative lesions affecting the integument and the mucosa of the gastrointestinal tract. Although the involvement of a CP strain is required for the development of MD, cattle acutely infected with highly virulent NCP strains may develop a clinical syndrome resembling mucosal disease (Hilbe et al. 2013).

The objective of this study was to characterize the anatomopathological and virological aspects of two distinct cases of mucosal disease in cattle from Santa Catarina state, southern Brazil.”

## Materials and methods

### Case characterization and anatomopathological analysis

The two cases included in this study were initially selected based on a history of suggestive clinical signs and anatomopathological findings compatible with mucosal disease. Animal 1 was a 24-month-old pregnant (five months of gestation) Hereford female from a beef cattle herd consisting of 11 animals raised under extensive management in county of Campo Belo do Sul, SC. The owner was a cattle trader, with a history of high animal turnover on the farm. No other animals in the herd were clinically affected. Animal 2 was a four-month-old Holstein male, originally acquired from a dairy farm and subsequently raised as a companion animal in county of Lages, SC.

Approximately 10 mL of whole blood were collected from each animal for serum separation, and serum samples were stored at − 80 °C for subsequent analyses. Both cattle were humanely euthanized in extremis in accordance with animal welfare guidelines and subsequently submitted to necropsy. Samples of liver, spleen, and thymus were also collected and stored at − 80 °C for complementary analyses. Tissue fragments from all major organs were fixed in 10% neutral buffered formalin, routinely processed for histopathology, and stained with hematoxylin and eosin (H&E).

### Immunohistological analysis

Formalin-fixed, paraffin embedded tissue sections from tongue, rumen and lymph node from animal 1 and rumen and intestine with Peyer’s patches from animal 2 underwent immunohistochemistry (IHC) for pestiviral antigen detection on unstained positive charged slides. The panpestiviral 15c5 antibody (dilution 1:10,000; E. Dubovi, Cornell University) targeting the glycoprotein gp48 (Erns) was used as described (Hilbe et al. 2007).

### Molecular analysis, sequencing, and phylogenetic analysis

Total RNA was extracted from serum samples using TRIzol™ LS Reagent (Thermo Fisher Scientific^®^), according to the manufacturer’s instructions. Complementary DNA (cDNA) was synthesized from 10 µL of RNA using the High-Capacity cDNA Reverse Transcription Kit (Thermo Fisher Scientific^®^), in a final reaction volume of 20 µL. The reverse transcription conditions followed the manufacturer’s recommendations for the kit and consisted of 25 °C for 10 min, 37 °C for 120 min, and 85 °C for 5 min, followed by holding at 4 °C (Marian et al. 2025).

Conventional PCR was performed to amplify a 288-bp fragment of the 5′ untranslated region (5′UTR) of pestiviruses, using primers 324 F (ATGCCCWTAGTAGGACTAGCA) and 326R (TCAACTCCATGTGCCATGTAC) (Vilček et al. 1994). PCR reactions were carried out in a final volume of 25 µL containing PCR buffer, MgCl₂, dNTPs, Platinum^®^ Taq DNA polymerase (Thermo Fisher Scientific^®^), primers, and template cDNA. According to the enzyme manufacturer’s instructions, the amplification conditions included an initial denaturation step at 94 °C for 2 min, followed by 35 cycles of denaturation at 94 °C for 30 s, annealing at 56.2 °C for 30 s, and extension at 72 °C, with a final extension step at 72 °C for 10 min and a holding temperature of 4 °C (Marian et al. 2025).

Amplification products were purified using a manual purification method and submitted to Sanger sequencing. The partial sequences obtained were edited and assembled using BioEdit™ software, version 7.2. Consensus sequences were subsequently compared with reference sequences available in GenBank using the BLASTn tool, in order to confirm genotypes and subgenotypes based on local sequence alignment (Marian et al. 2024, 2025).

For phylogenetic analysis, the sequences obtained in this study, together with reference pestivirus sequences previously deposited in GenBank, were aligned using the CLUSTAL W method (Thompson et al. 1994). The phylogenetic tree was constructed using the Maximum Likelihood method with 1,000 bootstrap replicates, and evolutionary distances were estimated using the Kimura 2-parameter model (Kimura 1980), using MEGA software, version 12 (Marian et al. 2024, 2025).

### Cell culture and viral isolation

Liver and spleen fragments were processed for viral isolation by mechanical maceration in supplemented DMEM, followed by homogenization, freeze–thaw cycles, centrifugation, and filtration of the supernatant. The resulting material was inoculated onto MDBK cell monolayers and incubated at 37 °C with 5% CO₂ for 2 h to allow viral adsorption. Subsequently, the inoculum was removed and replaced with DMEM containing 2% fetal bovine serum, and cultures were maintained for eight days under the same incubation conditions. Cultures were monitored daily for the detection of cytopathic effect (CPE), with complete medium replacement performed on day five. Negative control was maintained in parallel under identical conditions. At the end of the incubation period, both cells and supernatants were stored at − 80 °C for subsequent molecular confirmation of viral presence by RT-PCR (Wang et al. 2014).

## Results

### Case characterization and anatomopathological analysis

Both cattle exhibited chronic clinical manifestations and a history of poor productive performance. Animal 1 showed a clinical course of approximately 20 days, characterized by inappetence, progressive weight loss, sialorrhea, lameness, and diarrhea, as well as oral and cutaneous lesions. Animal 2 was underdeveloped and had a clinical course of approximately 30 days, marked by weight loss, intermittent diarrhea, dehydration, apathy, anorexia, and prolonged lateral recumbency.

**Fig. 1:**
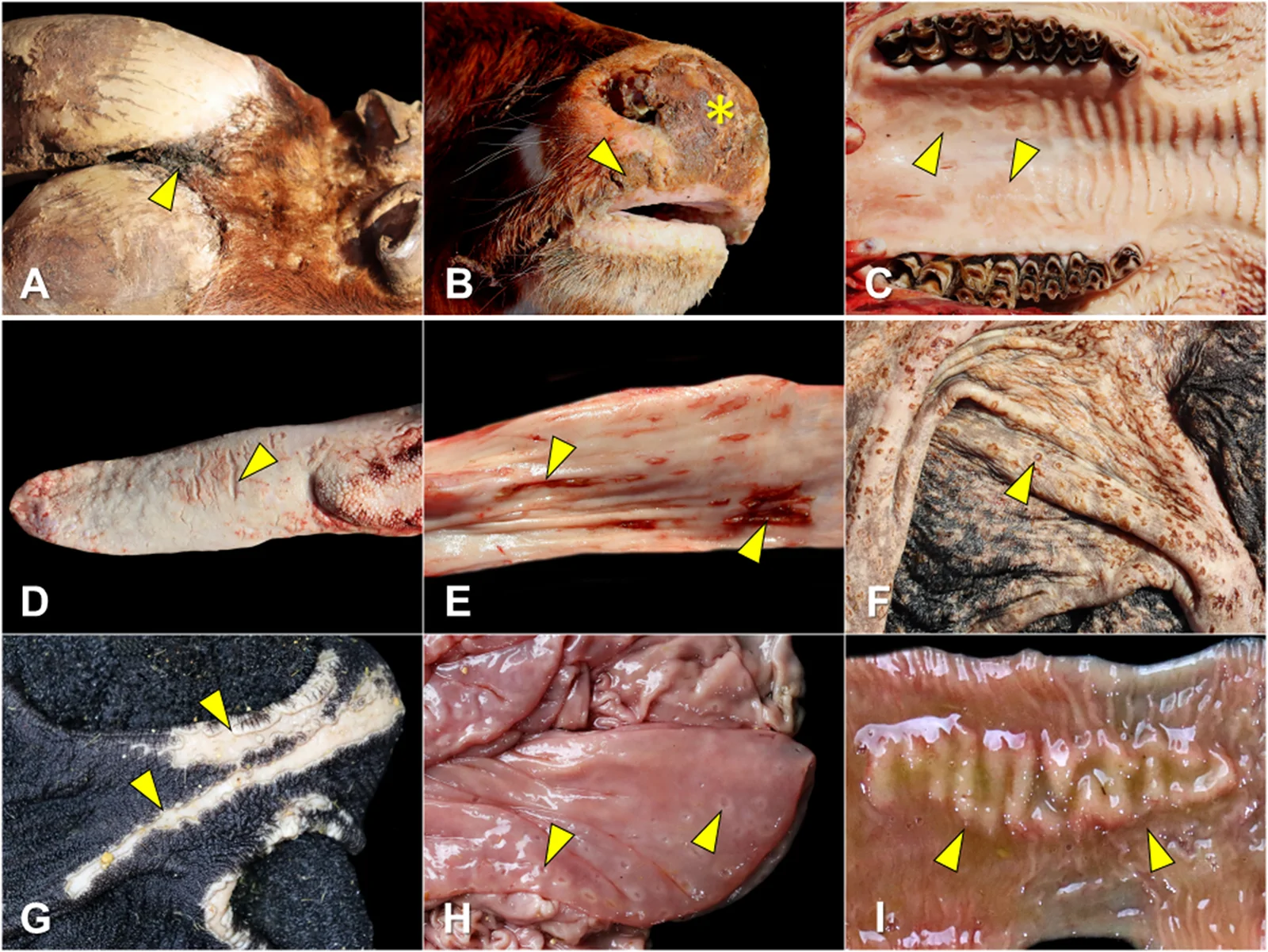
Gross lesions in cattle affected by mucosal disease (MD) associated with BVDV in the state of Santa Catarina, southern Brazil. Animal 1 – **(A)** Hull: ulceration up to 5 cm in diameter with crust formation affecting the interdigital skin (arrow) and coronary band. **(B)** Nasal planus: crust formation (arrow) and ulcerations up to 4 cm extending to the mucocutaneous junction (asterisk). **(C)** Hard palate: circular erosions measuring 1–3 cm on the mucosal surface (arrows). **(D)** Tongue: linear ulcerations measuring 0.5–2 cm on the dorsal surface (arrow). **(E)** Esophagus: linear erosions and ulcerations measuring 0.5–2 cm affecting the mucosa (arrows). **(F)** Rumen: circular erosions and ulcerations measuring approximately 0.5–1 cm on the mucosa, predominantly affecting the ruminal pillars (arrow). Animal 2 – **(G)** Rumen: linear ulcerations ranging from 5 to 15 cm in length in the region of the ruminal pillars (arrows). **(H)** Abomasum: pinpoint erosions up to 0.2 cm in diameter on the mucosa (arrows). **(I) Ileum**: prominent and enlarged Peyer’s patches (arrows)

Grossly, in Animal 1, the coronary band and interdigital regions of both thoracic and pelvic limbs exhibited ulcerative lesions measuring approximately 5 cm in diameter, with marked crust formation (Fig. 1a). At the nasal planus, there was accumulation of purulent exudate and crusts, in addition to ulcerative lesions up to 4 cm in diameter extending to the mucocutaneous junction (Fig. 1b). Within the oral cavity, circular erosions ranging from 1 to 3 cm were observed on the hard palate (Fig. 1c), along with linear ulcerations measuring 0.5 to 2 cm on the dorsal surface of the tongue (Fig. 1d). Along the esophagus, linear erosions and ulcerations measuring 0.5 to 2 cm extended from the cervical to the thoracic portion (Fig. 1e). In the rumen, multiple circular erosions and ulcerations measuring 0.5 to 1 cm were present on the mucosa, predominantly affecting the ruminal pillars (Fig. 1f). In the abomasum, pinpoint to stellate erosions measuring up to 0.2 cm in diameter were observed on the mucosal surface.

In Animal 2, gross examination revealed linear ulcerations in the rumen, ranging from 5 to 15 cm in length, predominantly affecting the ruminal pillars (Fig. 1g). In the abomasum, pinpoint erosions measuring approximately 0.2 cm in diameter were observed on the mucosal surface (Fig. 1h). In the small intestine, particularly in the ileal segment, Peyer’s patches were prominent, enlarged, and exhibited an edematous appearance (Fig. 1i).

**Fig. 2:**
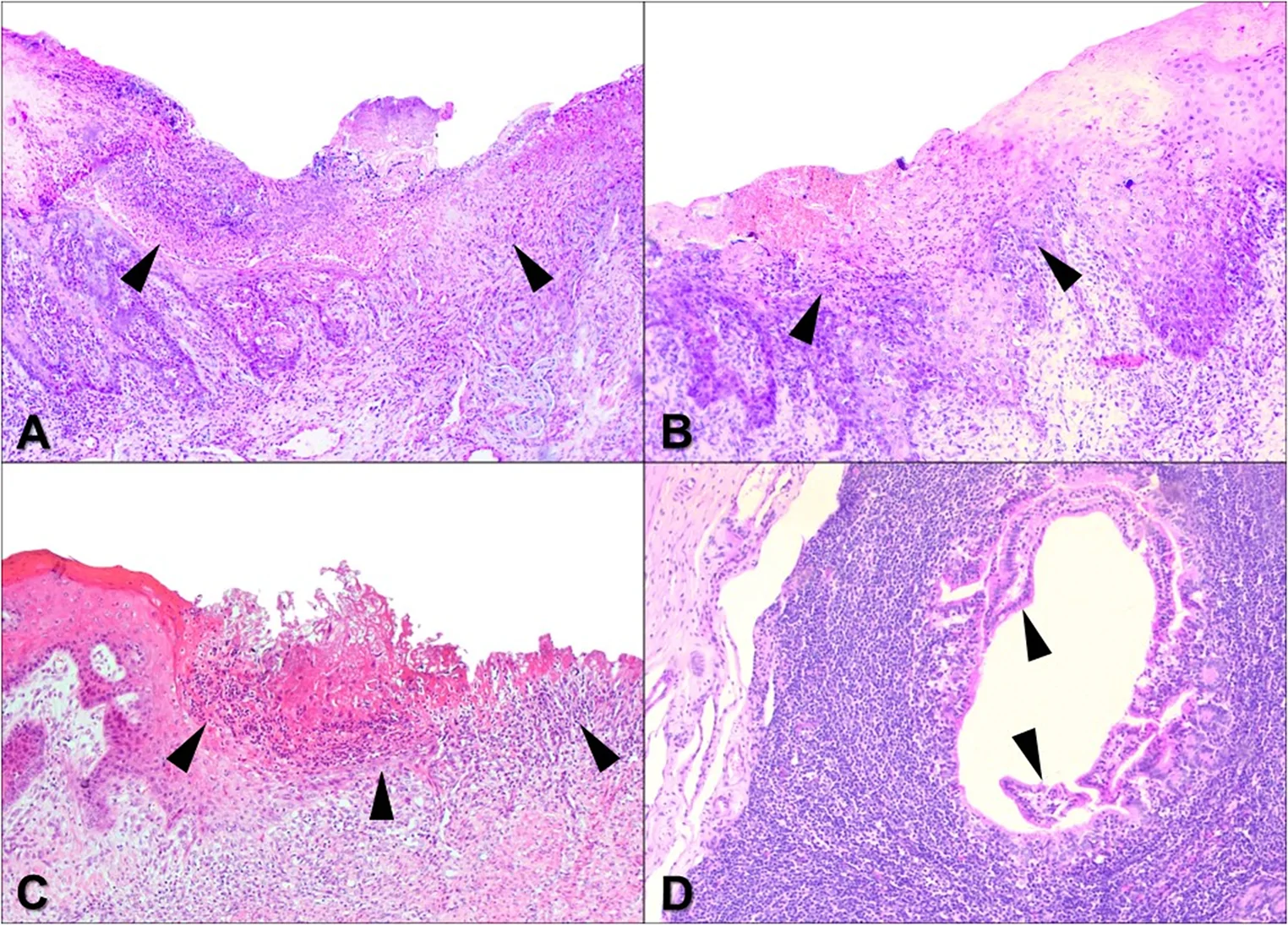
Histological lesions in cattle affected by mucosal disease (MD) in the Santa Catarina state, southern Brazil. Animal 1 **– (A)** Tongue: ulceration of the lining epithelium accompanied by infiltration of neutrophils, macrophages, lymphocytes, and plasma cells, along with necrotic debris and fibrinous exudation (arrows). **(B)** Esophagus: mucosal erosion associated with infiltration of neutrophils, macrophages, lymphocytes, and plasma cells (arrows). Animal 2 **– (C)** Rumen: mucosal ulceration accompanied by infiltration of neutrophils, macrophages, lymphocytes, and plasma cells and fibrinous exudation (arrows). **(D)** Ileum: in Peyer’s patches, loss of lymphofollicular architecture and formation of central cavitations within lymphoid follicles, internally lined by squamous epithelium and short microvilli (arrows)

Histopathological analysis of Animal 1 revealed ulcerative glossitis (Fig. 2a), erosive stomatitis, and erosive to ulcerative esophagitis (Fig. 2b), as well as erosive and ulcerative rumenitis. These lesions were characterized by loss of epithelial continuity, accompanied by infiltration of neutrophils, macrophages, lymphocytes, and plasma cells, along with necrotic debris and fibrinous exudation. Ulcerative and crusting dermatitis was observed in the interdigital region and coronary band, as well as ulcerative and crusting dermatitis affecting the nasal planum and mucocutaneous junction. These lesions were characterized by necrosis of the epidermis and dermis, associated with infiltration of neutrophils, macrophages, lymphocytes, and plasma cells, together with fibrinous exudation. Additionally, serocellular crusts composed of necrotic debris, fibrin, erythrocytes, and intact and degenerate neutrophils were present. In the abomasum, superficial mucosal necrosis was observed, accompanied by infiltration of macrophages, lymphocytes, and plasma cells.

In Animal 2, in addition to the histological lesions observed in the rumen (Fig. 2c) and abomasum previously described for Animal 1, ileal Peyer’s patches exhibited lymphoid follicles with poorly preserved architecture associated with marked central follicular depletion. This resulted in the formation of lymphocyte-depleted cavities, which were occasionally lined by a layer of squamous epithelial cells and filled with crypts and villi herniating through the submucosa (Fig. 2d).

### Immunohistological analysis

**Fig. 3:**
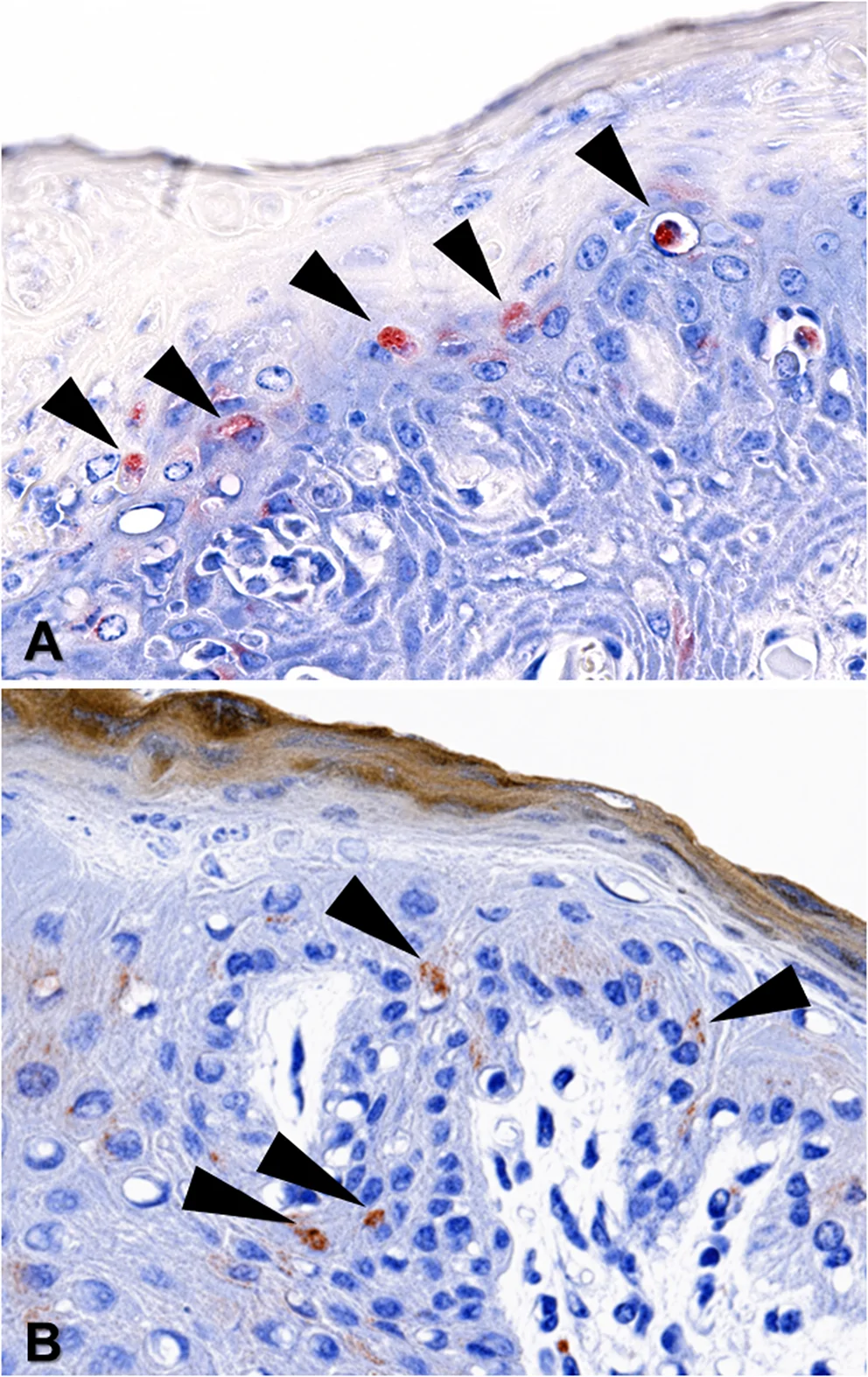
Immunohistochemical detection of pestiviral antigen 15c5, targeting the glycoprotein gp48 (Erns), in cattle affected by mucosal disease (MD) associated with BVDV in the Santa Catarina state, southern Brazil. Animal 1 (**A**) and Animal 2 (**B**) showed positive cytoplasmic staining in epithelial cells of the rumen (arrows)

Animal 1 and animal 2 showed positive cytoplasmic signal in the epithelial cells in the rumen, mostly near the ulcerative lesions (Fig. 3). Animal 1 showed also few positive epithelial cells in the tongue. Single media cells of the vessels in both animals were as well positive.

### Molecular analysis, sequencing, and phylogenetic analysis

Serum samples from both cattle tested positive for BVDV by RT-PCR. The sequences obtained through genetic sequencing demonstrated varying levels of similarity to pestivirus sequences previously deposited in GenBank (Table 1). The sequence obtained from Animal 1 (UDESC/11/2025), classified as BVDV-1e, showed greater than 97% similarity to Brazilian, European, and other South American sequences. The sequence derived from Animal 2 (UDESC/12/2025), classified as BVDV-1b, exhibited greater than 98% similarity to Brazilian, European, and Asian sequences.

**Table 1:** Comparative analysis of nucleotide sequence similarity between national and international BVDV-1 sequences deposited in GenBank and the sequences obtained from cattle with mucosal disease (MD) in Santa Catarina state, southern Brazil

Sequence	Genotype and subgenotype	Similar sequences	Origin	Degree of similarity
UDESC/11/2025 Animal 1	BVDV-1e	UDESC:05	Brazil	100%
		UDEC-COY13-17	Chile	
		bo228/02	Spain	
		21 − 07	France	
		LV/LF15/12	Brazil	99.5%
		LV/152/18		99.1%
		UDESC:01		98.2%
		UDESC:02		97.1%
UDESC/12/2025 Animal 2	BVDV-1b	BJ-2016	China	98.9%
		TR79	Turkey	98.8%
		UDESC/06/2025 UDESC/07/2025	Brazil	98.5%

**Fig. 4:**
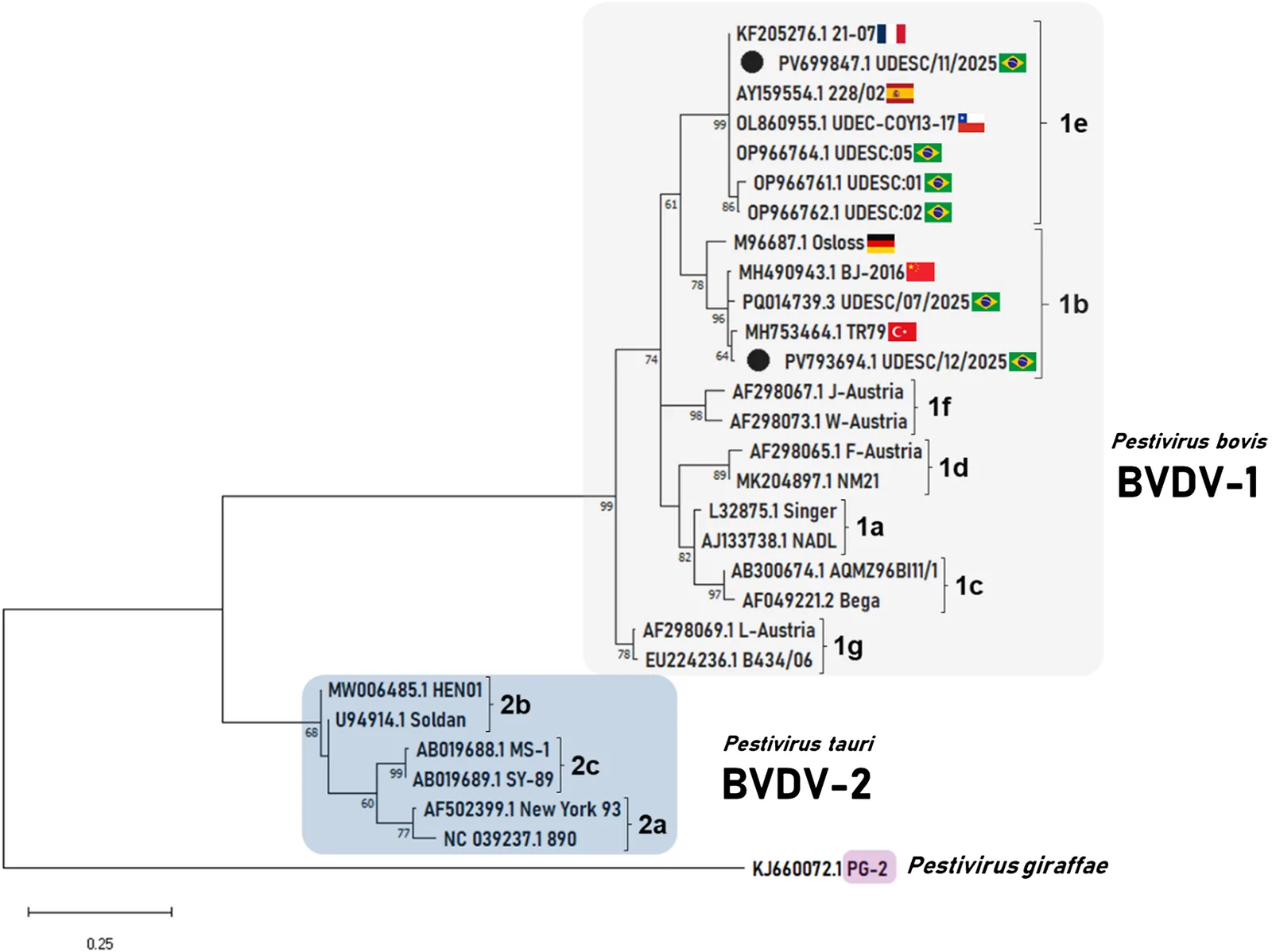
Phylogenetic tree based on the 5′UTR region of the pestivirus viral genome, including sequences obtained from two cattle with mucosal disease, as well as Brazilian and international sequences of Pestivirus bovis (BVDV-1), Pestivirus tauri (BVDV-2), and Pestivirus giraffae, analyzed using the Maximum Likelihood method with 1,000 bootstrap replicates under the Kimura 2-parameter model. The sequences identified in this study are indicated by a distinctive symbol(●)

The phylogenetic tree generated using sequences from different pestivirus species and subgenotypes previously deposited in GenBank, along with BVDV strains showing high similarity to the isolates obtained in this study, distributed the sequences into three distinct clades (Fig. 4). One clade corresponded to *Pestivirus bovis* (BVDV-1), within which the sequences from animals 1 and 2 were allocated. These sequences clustered, respectively, with BVDV-1e and BVDV-1b reference strains, confirming the genotype and subgenotype classification of each isolate. The remaining clades corresponded to sequences of *Pestivirus tauri* (BVDV-2) and *Pestivirus giraffae* (GPeV).

### Cell culture and viral isolation

**Fig. 5:**
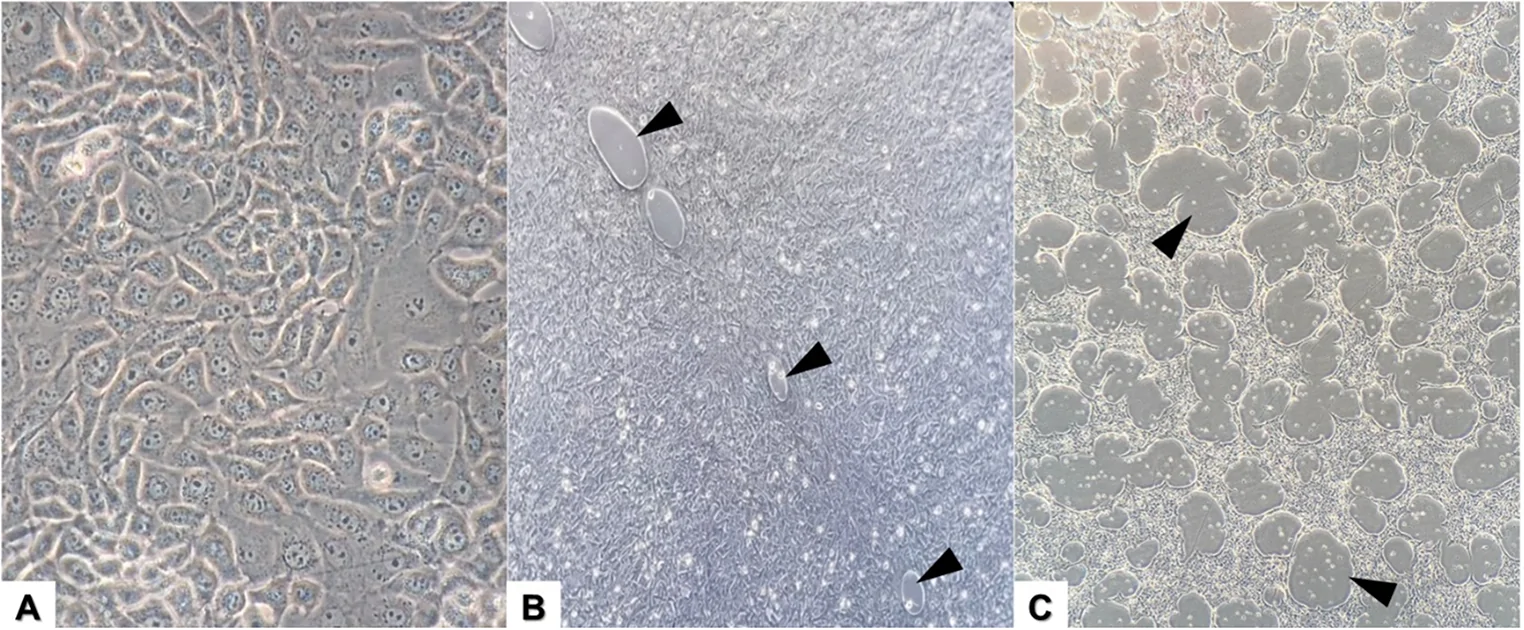
Cell culture and viral isolation in MDBK cells using samples from cattle infected with BVDV-1b and BVDV-1e in Santa Catarina state, southern Brazil. **A – Control culture**: intact cell monolayer. **Animal 1 **– (**B**): early cytopathic effect, characterized by the appearance of cellular plaques at 96 h post-inoculation (arrows). **Animal 2** – (**C**): intensification and diffusion of cellular plaques in the culture flask at 72 h post-inoculation

The samples were compared with negative control cell culture (Fig. 5a). For samples from Animal 1 (Fig. 5b), cytopathic effect (CPE) became evident at 96 h post-inoculation, characterized by the formation of small plaques associated with cellular vacuolization, which remained stable through 120 h. In samples from Animal 2 (Fig. 5c), CPE was observed as early as 48 h post-inoculation, with marked vacuolization and formation of small cellular plaques. By 72 h, plaques became diffuse and widely distributed, progressing to generalized cell lysis by 120 h.

## Discussion

Tegumentary and gastrointestinal lesions may be associated with severe acute BVDV infections; however, they occur predominantly in persistently infected animals in the form of mucosal disease, involving a variety of genotypes and subgenotypes (Bianchi et al. 2017, 2018). Lesions like those observed in cases of mucosal disease were associated with BVDV-1b, detected in Animal 2, in confined cattle in the United States, which exhibited extensive mucosal involvement, although occurring during an acute disease outbreak (Hessman et al. 2012). In Argentina, subgenotype 1b was also responsible for disease outbreaks in cattle presenting with generalized dermatitis (Odeón et al. 2003), in addition to two individual episodes of typical mucosal disease associated with cytopathic (CP) isolates, as observed in this animal (Pecora et al. 2014). In Brazil, there are no reports linking BVDV-1b to classical cases of mucosal disease; this subgenotype has been associated with an outbreak presenting gastrointestinal lesions like those observed in the present study, but without confirmation of involvement of cytopathic (CP) strains (Lunardi et al. 2008).

Regarding the involvement of BVDV-1e in the development of mucosal disease, searches conducted on scientific research platforms such as PubMed and Google Scholar revealed a single study from Switzerland associating this subgenotype with cutaneous and gastrointestinal lesions in three cattle, with a typical mucosal disease presentation observed in only one of the animals (Hilbe et al. 2013). There is evidence linking BVDV-1e primarily to reproductive failures, as it is the main subgenotype detected in stillborn calves and aborted fetuses in studies conducted in Switzerland and Santa Catarina (Brazil). This suggests that BVDV-1e has a greater detrimental impact on prenatal and perinatal losses compared to its involvement in the development of other diseases, such as MD (Bachofen et al. 2010; Marian et al. 2024).

The lesions observed in the cattle of this study affected multiple regions of the digestive tract and were associated with cytopathic (CP) isolates, confirming the occurrence of MD (Hilbe et al. 2013). The extensive distribution of digestive lesions observed in Animal 1, with ulcerations extending from the nasal planum and oral cavity to the abomasum, without intestinal involvement, was like that reported in an outbreak in Rio Grande do Sul. Additionally, the animals developed crusting dermatitis, although affecting the axillary, abdominal, and inguinal regions (Bianchi et al. 2017) affecting the axillary, abdominal, and inguinal regions (Bianchi et al. 2017), this contrasts with the present study, in which the animal exhibited lesions on the hooves. In another outbreak involving calves in Mato Grosso, digestive lesions affected the same organs but also involved the intestines, while the skin showed interdigital crusting dermatites (Cruz et al. 2018). Like Animal 1, heifers in Argentina exhibited tegumentary lesions in the interdigital space and coronary band associated with gastrointestinal manifestations, but with ulcerations confined to the oral cavity and esophagus (Odeón et al. 2003).

There was no involvement of the integument in Animal 2, in which lesions were restricted to the digestive system (Lunardi et al. 2008; Hessman et al. 2012). This pattern of lesions has also been reported in an outbreak involving *Pestivirus braziliense* (HoBi-like), which presented lesions in the oral cavity, tongue, esophagus, and omasum, while the rumen and intestines remained unaffected (Weber et al. 2016). The pattern of digestive lesion distribution observed in Animal 2 appears unusual, as in all reports associated with BVDV in which the rumen, abomasum, or Peyer’s patches are involved, other regions, such as the oral cavity and esophagus, are typically also affected (Odeón et al. 2003; Cruz et al. 2018).

The variation in lesion distribution observed in the cattle of this study may be related to individual factors, such as immune status, as well as the duration of disease progression (Brodersen 2014). In acute cases, lesions tend to appear in more superficial regions and are therefore more evident in the oral cavity and esophagus, as observed in Animal 1 (Bianchi et al. 2017). The pronounced ruminal ulcerations and involvement of lymphoid tissue in Peyer’s patches suggest a subacute to chronic course, since, as the disease progresses, mild lesions may heal, whereas deeper lesions persist or worsen (Liebler-Tenorio et al. 2000; Bianchi et al. 2017, 2018).

As with the gross findings, the histological lesions observed in the integument and digestive system of these cattle are typical and frequently associated with BVDV infection (Odeón et al. 2003; Bianchi et al. 2017, 2018; Cruz et al. 2018). Along the digestive mucosa of Animal 1 and 2, erosions and ulcerations characterized by epithelial necrosis were the main lesions and were generally associated with mixed inflammatory infiltrates and fibrinous exudation (Hessman et al. 2012; Bianchi et al. 2017). In the hoof region of Animal 1, histologically, in addition to crust formations, the epidermis was ulcerated, as is frequently reported in cattle with lesions in the interdigital space and coronary band (Odeón et al. 2003; Bianchi et al. 2018).

Characterizing lymphoid lesions represents a challenge in natural cases of mucosal disease, as such tissue alterations are predominantly assessed under experimental conditions (Teichmann et al. 2000; Liebler-Tenorio et al. 2000). The collapse of lymphoid follicles and loss of tissue architecture, manifested as central follicular cavities in Animal 2, are typical of advanced mucosal disease and are associated with failure of tissue regeneration manifested as central follicular cavities in Animal 2, are typical of advanced mucosal disease and are associated with failure of tissue regeneration (Teichmann et al. 2000). Severe depletion accompanied by loss of follicular structure in Peyer’s patches has also been observed experimentally in late-onset mucosal disease, when there is a delayed progression following infection with a cytopathic (CP) strain (Liebler-Tenorio and Pohlenz 1997; Liebler-Tenorio et al. 2000). Similar lesions have been reported in natural infections with BVDV-1b, although with greater severity in portions of the large intestine (Lunardi et al. 2008), unlike in this animal, in which only ileal Peyer’s patches were affected.

The intracytoplasmic immunolabeling observed in epithelial cells of the gastrointestinal tract of these cattle is a finding described in BVDV infections (Cruz et al. 2018), particularly in areas adjacent to regions of damaged mucosa (Silva Silveira et al. 2020). Labeling using different antibodies, including the 15c5 antibody employed in this study, can be observed in sections of various tissues from BVDV-infected animals, with variable intensity, being particularly evident in digestive tract organs lined by keratinized stratified epithelium, such as the tongue and rumen (Hilbe et al. 2013; Bianchi et al. 2017).

Regarding the geographic distribution and phylogenetic relationships of the BVDV isolates identified in this study, the sequence UDESC/11/2025, obtained from Animal 1 and classified as BVDV-1e, showed high similarity to sequences previously detected in Santa Catarina, including samples from aborted fetuses (Marian et al. 2024) and follicular fluid (Silveira et al. 2017). This pattern highlights the regional circulation of genetically related strains, suggesting the local maintenance of lineages with a common ancestry over time for this subgenotype, which in Brazil occurs exclusively in Santa Catarina (Mosena et al. 2022). Furthermore, all BVDV-1e sequences detected in the country, including those from this study, exhibit high similarity to the Spanish isolate bo228/22 (Hurtado et al. 2003) and the French isolate 21/07. Although highly similar sequences have also been reported in Chile, there are no records of cattle movement between the Andean country and Brazil (Hugues et al. 2023) making direct introduction from Europe to South American countries via live cattle importation a more likely scenario (Mosena et al. 2022).

In contrast, the sequence UDESC/12/2025, classified as BVDV-1b and obtained from Animal 2, showed high genetic similarity to sequences previously identified in an outbreak in a beef herd in the Serra Catarinense region (Marian et al. 2025), possibly facilitated by the proximity of the case origins and local cattle trade (Qi et al. 2019). Additionally, the sequence exhibited high similarity with international isolates. Its resemblance to sequence TR79, obtained from imported cattle in Turkey (Alpay et al. 2018), may be related to live animal trade, as thousands of cattle from southern Brazil have been exported to Middle Eastern countries in recent years (Ermgassen et al. 2020). Regarding similarity with Chinese isolates, a possible ancestral origin from Asia should be considered, from where there was extensive importation of Zebu cattle to Brazil until the 1960s, coinciding with the emergence and early diversification of BVDV-1b in Brazil, estimated around 1950 (Weber et al. 2021; Mosena et al. 2022).

## Conclusion

This study documents the occurrence of erosive and ulcerative lesions in the gastrointestinal tract and/or integument of two cattle infected with BVDV, without a direct epidemiological link. The detection of the cytopathic biotype in both animals constitutes the first report of typical mucosal disease associated with the BVDV-1b and BVDV-1e subgenotypes in Brazil. These findings reinforce the importance of investigating atypical or severe clinical cases compatible with mucosal disease. Considering the absence of a national program specifically dedicated to BVDV surveillance and control in Brazil, the investigation and molecular characterization of suspected cases may contribute to a better understanding of BVDV epidemiology and viral diversity in the country.

## Data Availability

Not applicable.
